# Clinicopathological Spectrum of B-Cell Non-Hodgkin Lymphoma in Pakistan Population: A Single-Center Study

**DOI:** 10.7759/cureus.34298

**Published:** 2023-01-28

**Authors:** Rashida Saleem, Anila Chughtai, Ghazi Zafar, Omar Chughtai, Saira Javeed, Akhtar S Chughtai

**Affiliations:** 1 Histopathology, Chughtai Institute of Pathology, Lahore, PAK

**Keywords:** b-cell non-hodgkin lymphoma, diffuse large b-cell lymphoma, chronic lymphocytic leukemia/small lymphocytic lymphoma, precursor b-cell lymphoblastic lymphoma, burkitt lymphoma

## Abstract

Background

B-cell non-Hodgkin lymphoma (NHL) is a common malignancy worldwide and in the Pakistani population. In our population, there was limited information regarding the clinicopathological characteristics of B-cell NHL. This study assessed the disease spectrum and most prevalent subtypes of B-cell NHL.

Methodology

An analysis of 548 cases was conducted in this cross-sectional study between January 2021 and September 2022, using a non-probability consecutive sampling approach. Patient age, gender, site of involvement, and diagnosis were documented according to the 5th edition of the World Health Organization (WHO) Classification of Tumors of Hematopoietic and Lymphoid Tissue, published in 2018. Data were entered and analyzed using Statistical Product and Service Solutions (SPSS) (IBM SPSS Statistics for Windows, Version 26.0, Armonk, NY).

Results

The mean age of the patients was 47.73±20.44 years. There were 369 males (67.34%) and 179 females (32.66%). The most prevalent type of B-cell NHL was diffuse large B-cell lymphoma (DLBCL) (58.94%), followed by chronic lymphocytic leukemia/small lymphocytic lymphoma (CLL/SLL) (13.14%), Burkitt lymphoma (9.85%), and precursor B-cell lymphoblastic lymphoma (5.11%). In contrast to low-grade B-cell NHL (22.99%), high-grade B-cell NHL was more common (77.01%). Nodal involvement was observed in 62.04% of cases. The cervical region was the most common nodal site of involvement (62.04%), and the gastrointestinal tract (GIT) was the most common extranodal site (48.29%).

Conclusion

The incidence of B-cell NHL is higher in older age groups. The most common nodal site was the cervical region, whereas the extranodal site was the GIT. The most reported subtype was DLBCL, followed by CLL/SLL, and Burkitt lymphoma. The prevalence of high-grade B-cell NHL is higher than that of low-grade B-cell NHL.

## Introduction

Lymphoma is a neoplasm of the lymphocytes. There are two main subgroups of lymphomas: Hodgkin lymphoma (HL) and non-Hodgkin lymphoma (NHL). NHL is further categorized into B-cell and T-cell types. B-cells are subcategorized into low- and high-grade, with different genetic and prognostic features [1,2].

Over the past few decades, cancer incidence has increased by approximately 30% worldwide [3], and NHL accounts for approximately 4-6% of all cancers. In the United States, NHL ranks eighth in cancer-related mortality [4]. Pakistan is included in the lymphoma belt, which stretches from Southeast Asia to the Middle East and Northern Africa. There has been an alarming increase in lymphoma cases in Pakistan in the past few decades [5,6].

From 1995 to 2002, the age-standardized incidence rate (ASIR) of lymphomas in the Karachi Cancer Registry increased from 5.3 to 8.4 per 100,000 for males and 4.1 to 6.5 per 100,000 for females [6]. NHL is the third most common malignancy among males and the sixth most common among females [7].

This study assessed the clinicopathological features of B-cell NHL in the Pakistan population.

## Materials and methods

Study design

A cross-sectional study using non-probability consecutive sampling was conducted between January 2021 and September 2022 at the Chughtai Institute of Pathology with Institutional Review Board approval (CIP/IRB/1123). It is one of the largest private laboratories in Pakistan that accepts samples from all over the country. Chughtai Laboratories operate in multiple locations throughout the country. Because of its geographical location in the middle of the second largest city in Pakistan, approximately 70% of the total number of patients attending this clinic are residents of different areas of Pakistan.

Inclusion criteria

According to the World Health Organization (WHO) Classification of Tumors of Hematopoietic and Lymphoid Tissues, all diagnosed cases of B-cell NHL were included in the study. In this study, individuals of both genders, regardless of their age, were included.

Exclusion criteria

The study excluded biopsies with poor preservation, tru-cut biopsies that were inconclusive for diagnosis, unclassifiable lymphomas, cytological specimens, as well as bone marrow biopsies.

Data collection

The data for this study were retrieved from the archive of the institute using an electronic data system (Nexus Pro). Data regarding patient age, gender, location of involvement, and subtypes of B-cell NHL were also documented. The specific subtypes of the tumor were accompanied by specific information regarding the type of tumor, its location, and whether it had been described as a nodal or extra-nodal variant.

Statistical analysis

Statistical Package for the Social Sciences (SPSS) version 26.0 (IBM Corp., Armonk, NY, USA) was used to analyze the data. Qualitative variables were presented as frequencies and percentages. Quantitative variables were presented as mean and standard deviation. A chi-square test was applied, and a p-value of less than 0.05 was considered statistically significant.

## Results

**Figure 1:**
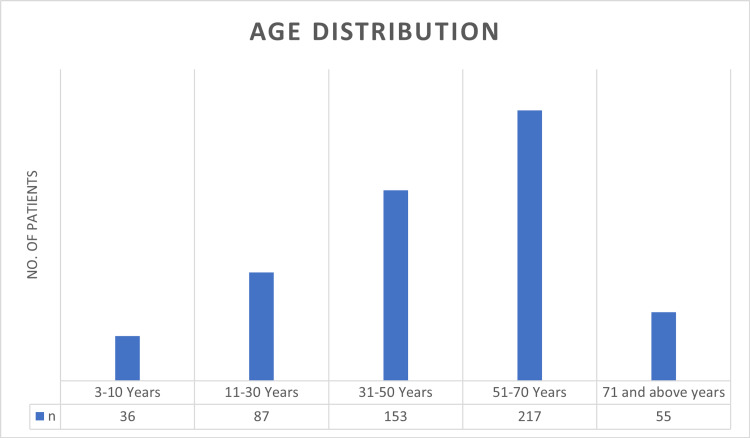
Age distribution

In total, 548 patients were included in this study. The mean patient age was 47.73±20.44 years (range 3-95 years). The study included 369 males (67.34%) and 179 females (32.66%). In males, the mean age was 47.92±20.98 years (range 4-95 years). In females, the mean age was 47.34±19.32 years (range 3-92 years). The distribution of age groups is shown in Figure 1.

**Figure 2:**
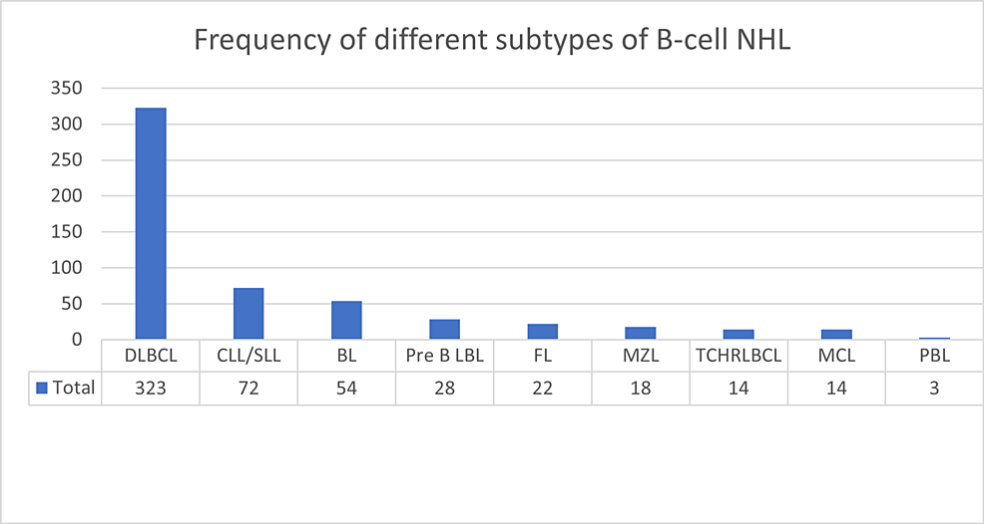
Frequency of different subtypes of B-cell NHL NHL: non-Hodgkin lymphoma, DLBCL: diffuse large B-cell lymphoma, CLL/SLL: chronic lymphocytic leukemia/small cell lymphoma, BL: Burkitt lymphoma, Pre B LBL: precursor B-cell lymphoblastic lymphoma, FL: follicular lymphoma, MZL: marginal zone lymphoma, TCHRLBCL: T-cell/histiocyte-rich large B-cell lymphoma, MCL: mantle cell lymphoma, PBL: plasmablastic lymphoma

There were 369 males (67.34%) and 179 females (32.66%). In total, there were 323 (58.94%) cases of diffuse large B-cell lymphoma (DLBCL), 72 (13.14%) cases of chronic lymphocytic leukemia/small lymphocytic lymphoma (CLL/SLL), 54 (9.85%) cases of Burkitt lymphoma (BL), 28 (5.11%) cases of precursor B-cell lymphoblastic lymphoma (pre-B LBL), 22 (4.01%) cases of follicular lymphoma (FL), 18 (3.28%) cases of marginal zone lymphoma (MZL), 14 (2.55%) cases of T-cell/histiocyte-rich large B-cell lymphoma (TCHRLBCL), 14 (2.55%) cases of mantle cell lymphoma (MCL), and three (0.55%) cases of plasmablastic lymphoma (PBL). The frequencies of different subtypes of B-cell NHL are shown in Figure 2 and the frequencies of different subtypes of B-cell NHL according to gender are shown in Table 1.

**Table 1:** Subtypes of B-cell NHL according to gender NHL: non-Hodgkin lymphoma

Type	Females n (%)	Males n (%)	Total n (%)
Diffuse large B-cell lymphoma	121 (22.08%)	202 (36.86%)	323 (58.94%)
Chronic lymphocytic leukemia/small cell lymphoma	12 (2.19%)	60 (10.95%)	72 (13.14%)
Burkitt lymphoma	13 (2.37%)	41 (7.48%)	54 (9.85%)
Precursor B-cell lymphoblastic lymphoma	12 (2.19%)	16 (2.92%)	28 (5.11%)
Follicular lymphoma	7 (1.28%)	15 (2.74%)	22 (4.01%)
Marginal zone lymphoma	6 (1.09%)	12 (2.19%)	18 (3.28%)
T-cell/histiocyte-rich large B-cell lymphoma	4 (0.73%)	10 (1.82%)	14 (2.55%)
Mantle cell lymphoma	3 (0.55%)	11 (2.01%)	14 (2.55%)
Plasmablastic lymphoma	1 (0.18%)	2 (0.36%)	3 (0.55%)
Grand Total	179 (32.66)	369 (67.34)	548 (100)

High-grade B-cell NHL accounted for 422 cases (77.01%) and low-grade B-cell NHL accounted for 126 cases (22.99%). The cervical region was the most common nodal site of involvement (n=149, 43.82%), followed by the inguinal region (n=65, 19.12%), axillary region (n=58, 17.06%), abdominal region (n=37, 10.88%), and other miscellaneous nodal sites (n=31, 9.12%).

The gastrointestinal tract (GIT) (n=99, 48.29%) was the most common site of extranodal involvement, followed by the head and neck (n=25, 12.20%), testes (n=20, 9.76%), vertebrae (n=14, 6.83%), mediastinum (n=13, 6.34%), soft tissue (n=13, 6.34%), central nervous system (n=8, 2.44%), and other miscellaneous extranodal sites (n=16, 7.80%). The distributions of nodal and extranodal site involvement are shown in Table 2.

**Table 2:** Distribution of nodal and extranodal sites of involvement

Site (Nodal)	n (%)	Site (Extranodal)	n (%)
Cervical region	149 (43.82%)	Gastrointestinal tract	99 (48.29%)
Inguinal region	65 (19.12%)	Head and neck	25 (12.20%)
Axillary region	58 (17.06%)	Testes	20 (9.76%)
Abdominal region	37 (10.88%)	Vertebrae	14 (6.83%)
Miscellaneous	31 (9.12%)	Mediastinum	13 (6.34%)
-	-	Soft tissue	13 (6.34%)
-	-	Central nervous system	8 (2.44%)
-	-	Miscellaneous	16 (7.80%)
Total	340 (62.04%)	Total	208 (37.96%)

## Discussion

NHL is the seventh most common and sixth most deadly malignancy worldwide. According to recent data in 2018, an estimated 509,600 new cases of NHL were diagnosed globally, comprising 2.8% of worldwide cancer diagnoses, and an estimated 248,700 global deaths were attributable to NHL, accounting for 2.6% of all oncological mortalities. An estimated 77,200 new cases of NHL were diagnosed in 2020 in the US, accounting for 4.3% of cancer diagnoses [3].

NHL represents a wide spectrum of illnesses, ranging from indolent to aggressive malignancies [8]. Precise diagnosis of NHL requires pathological examination and classification according to the WHO Classification of Tumors of Hematopoietic and Lymphoid Tissues [9,10]. The estimated NHL incidence rates may reflect the infectious origin of the disease in Africa, South America, and Asia. Accumulating evidence suggests that Epstein-Barr virus (EBV) is associated with NHL etiology [11]. The incidence of these cases is higher among the elderly due to age-related changes in the immune system and prolonged latency of EBV infection [12].

The mean age in our study was 47.73±20.44 years. The mean age of all NHL patients in a study by Shahid et al. [13] was 46.31+18 years. According to Mahmood et al., the mean age of the patients was 49.7 years [14]. Our results are similar to those of these studies. There were 369 males and 179 females in our study, with a male-to-female ratio of 2.06:1. The male-to-female ratio was reported by Aslam et al. [15] in patients with NHL is close to ours: 1.83:1.

In our study, the prevalence of DLBCL was 58.94%, followed by that of CLL/SLL (13.14%). The results of our study are similar to those of Shahid et al. and Devi et al., who reported DLBCL as the most common lymphoma [13,16]. The etiology of lymphoma in Pakistan remains unknown. Based on ethnicity, environment, and other neighborhood characteristics, the literature does not draw any conclusions about why DLBCL is most common in Asian populations. Many genetic factors may contribute to the development of lymphoma, including gene rearrangement, protein overexpression, and p53 overexpression [17]. In Western countries, the prevalence of lymphoma subtypes varies owing to genetic differences, viral effects, and environmental factors. Our study found that the cervical region was the most frequently involved (27.19%).

A review by Laurent et al. [17] found that cervical lymph nodes were the most frequently involved sites (43.82%). Shahid et al. [13] also reported that cervical lymph nodes were the most frequently involved sites. These results are consistent with those of the present study. Jayanta et al. found that the GIT is the most common extranodal site for NHL involvement, based on a retrospective analysis of pictorial positron emission tomography (PET) scans from different regions [18]. Our study showed similar results to those reported in other studies [13-17].

Limitations

Our study had several limitations, including the fact that approximately one-third of our patients in Pakistan presented with stage IV disease, as described by Aslam et al. [15]. This could be due to the low socioeconomic status and lack of awareness among people in the northern and southern areas of Pakistan, including Balochistan. Bone marrow specimens were not included; therefore, staging of most lymphomas, such as CLL/SLL, could not be performed. The central registry in Pakistan has not been provided with appropriate data, and it is impossible to estimate the absolute number of lymphoma cases based on data gathered from leading tertiary care local registries that provide information on lymphoma. It was a single-center study without any collaboration with local central registries, and the number of cases included was not enough to highlight the various etiological agents responsible for the increasing number of lymphoma cases in Pakistan.

## Conclusions

DLBCL is the most commonly reported subtype, followed by CLL/SLL, Burkitt’s lymphoma, and pre-B LBL. Less than 4% of cases were accounted for by other disease subtypes. There was a high rate of nodal involvement in the cervical region, whereas extranodal involvement was most common in the GIT. There has been a significant increase in the incidence of lymphoma among older people, particularly those aged greater than 60 years. High-grade B-cell NHL is more common than low-grade B-cell NHL. Among low-grade B-cell NHL, CLL/SLL is the most common, followed by FL and MZL. DLBCL is more common among high-grade lymphomas, followed by Burkitt’s lymphoma, and pre-B LBL.
